# Development of antimicrobial nanoemulsion edible coating of xanthan gum incorporated with pomelo peel extract for cheese preservation

**DOI:** 10.1016/j.fochx.2025.102692

**Published:** 2025-06-25

**Authors:** Manisha Joshi, Gurvendra Pal Singh, Ipsheta Bose, Tianxi Yang, Azadeh Babaei, Somesh Sharma, Krishna Aayush

**Affiliations:** a School of Bioengineering and Food Technology, Shoolini University, Bajhol, Distt Solan 173229, India; b Department of Food Technology School of Engineering and Technology, Jaipur National University, Jaipur, Rajasthan 302017, India; c Food, Nutrition and Health, Faculty of Land and Food Systems, The University of British Columbia, Vancouver, BC V6T 1Z4, Canada; d Department of Chemistry, Ka.C., Islamic Azad University, Karaj, Iran; e MS Swaminathan School of Agriculture, Shoolini University, Bajhol, Distt Solan 173229, India; f Department of Food Science and Technology, Graphic Era (Deemed to be University), Dehradun, Uttarakhand 248002, India

**Keywords:** Active nanoemulsion, Cottage cheese, Edible coatings, Pomelo peel extract, Shelf-life extension, Waste valorisation

## Abstract

The increasing demand for natural food preservation highlights the potential of plant-based edible coatings. This study developed an active nanoemulsion edible coating using xanthan gum (XG) infused with pomelo peel extract (PPE) for paneer preservation. Pomelo peel extracts prepared using methanol, hexane, and dichloromethane showed methanol extract had the highest phenolic (177.06 ± 0.08 mg GAE/g), flavonoid (19.20 ± 0.12 mg RU/g), and antioxidant activity (70.49 ± 0.23 % DPPH inhibition). Antimicrobial activity was confirmed against spoilage organisms, with the highest inhibition zones for *Shigella boydii* (28.3 ± 1.1 mm), *Bacillus cereus* (27.6 ± 0.5 mm), and *Rhizopus stolonifer* (23 ± 2 mm). The nanoemulsion (1.5 % Tween 80, 0.2 % XG, 2 % PPE) was applied to paneer, significantly reducing microbial counts (yeast and mold: 3.31 ± 0.04 log₁₀ CFU/mL; total plate: 3.52 ± 0.01 log₁₀ CFU/mL) over 15 days at 4 °C. It also reduced weight loss (9.62 ± 0.47 %), maintained pH (4.53 ± 0.2), limited lipid hydrolysis (0.81 ± 0.09 %), and preserved acidity (0.99 ± 0.06 %) and sensory quality. This approach supports sustainable food preservation and citrus peel valorisation.

## Highlights

• Pomelo peel extract (PPE) rich in phenolics and flavonoids with antioxidant power.
• PPE nanoemulsion made using Tween 80 and xanthan gum for food-grade coatings.
• PPE shows strong antimicrobial action against spoilage bacteria and fungi.
• Paneer shelf life improved with PPE-based nanoemulsion edible coating.
• Citrus peel waste valorised via sustainable PPE coating for food preservation.

## 1 Introduction

The food industry faces major challenges in preserving dairy products because they are highly perishable and prone to microbial degradation caused by spoilage and pathogenic microorganisms such as *Pseudomonas* spp., *Escherichia coli*, *Listeria monocytogenes*, *Salmonella* spp., *Bacillus cereus*, and fungus like *Aspergillus niger, Candida albicans, and Rhizopus stolonifer* (Martin et al., 2021)*.* The cellular structure, high moisture content, and vulnerability to spoiling bacteria significantly reduce their shelf life, resulting in large losses. Indian cottage cheese (ICC)/ paneer, a widely consumed dairy product in South Asia, poses major preservation challenges. It is usually prepared from buffalo milk by heat and acid coagulation and has a firm but spongy texture, a marble-white color, with some degree of sweetness and acidity. Its shelf life is, however, limited to 3 days at ambient temperature and up to 6 days at 10 °C because of quality degradation (Alam et al., 2024). The main cause of spoiling, which results in rancid flavors due to decreased quality, is lipid oxidation, particularly in the polyunsaturated fatty acids of ICC/ paneer (Goyal & Goyal, 2016). Conventional preservation techniques, such as refrigeration and chemical treatments, aim to slow down microbial growth and delay spoilage (Chaudhary et al., 2025; Prasad et al., 2022). In addition, synthetic antioxidants such as butylated hydroxyanisole (BHA) and tertiary butylhydroquinone (TBHQ) are widely utilized to prevent lipid oxidation (Gutiérrez-del-Río et al., 2021). Preservation methods, including vacuum-sealing and modified atmosphere packaging (MAP) using inert gases like CO₂ and N₂, along with multilayer polyethylene films, have been used to maintain product quality (Gao et al., 2023; Roman et al., 2016). However, synthetic antioxidants are effective in delaying lipid oxidation and extending shelf life, but raise concerns regarding potential health risks (Khezerlou et al., 2022; Zhang et al., 2023). Moreover, conventional plastic-based packaging contributes to environmental pollution due to its non-biodegradable nature, prompting increased regulatory measures and greater consumer demand for sustainable alternatives. Additionally, vacuum and MAP technologies, although effective, require costly equipment and controlled handling conditions, limiting their practicality for small-scale producers. Considering these limitations, there is growing interest in natural, biodegradable alternatives such as edible coatings, which provide a promising, sustainable solution like biodegradable materials, active packaging, and intelligent packaging technologies (Khalid et al., 2024). Among these, active packaging, like edible coatings with antimicrobial properties, has emerged as a new approach to food preservation, particularly for dairy products like Indian cottage cheese (ICC)/paneer (Khalid et al., 2024; Joshi et al., 2024). Biopolymer-based edible coatings derived from plant materials such as polysaccharides (xanthan gum, alginates), proteins (casein, whey), and lipids can be called a sustainable solution as they reduce reliance on plastic packaging, degrade naturally without contributing to pollution, and minimize the use of synthetic additives. These biopolymer-based edible coatings form a barrier that limits moisture loss, lowers respiration rates, and prevents microbiological growth by the controlled release of active bioactive chemicals encapsulated by NE technology (Aayush et al., 2024, Singh, Aayush and Yang, 2023; Azam et al., 2024). This mechanism satisfies consumer preferences for natural preservation techniques while extending shelf life without sacrificing food quality (Sivashankari & Sadvatha, 2024). Additionally, this coating requires less energy and equipment than other conventional preservation techniques. An innovative method for creating active edible packaging is NE, which encapsulates antibacterial agents (Singh et al., 2022). The encapsulated bioactive compounds are slowly released through this complex system, enabling a dual-action mechanism that effectively suppresses microbial growth and extends the product's shelf life within specified timeframes (Sharma et al., 2022). This sustainable approach, utilizing NE technology, not only preserves the product's quality but also establishes a dynamic equilibrium in the release of antimicrobial agents targeting pathogens such as *Salmonella*, *Escherichia coli*, *Staphylococcus aureus*, *Bacillus cereus*, *Campylobacter*, *Clostridium perfringens*, *Aspergillus niger* and others (Ghazy et al., 2023). However, incorporating an active bioactive agent, primarily derived from various herbal and plant sources, is crucial for developing functional NE systems. Dairy products, such as cheese, lack phytochemicals to inhibit pathogens, which further reduces their shelf life. Incorporating plant extract-based active nanoemulsions (NE) can enhance shelf life without chemical preservatives and fortify the food with antioxidant compounds (Aayush et al., 2022, Aayush et al., 2025). This approach also aligns with the growing demand for natural, clean-label preservation strategies. Additionally, valorisation of agro-food waste such as fruit peels, seeds, pomace, and vegetable residues provide a sustainable source to extract bioactive compounds like antioxidants and antimicrobials, which can be used to develop sustainable packaging materials (Tahir et al., 2023). Extraction methods, like solvent extraction, supercritical fluid extraction, and green extraction, are used to obtain valuable compounds from agro-food waste. This approach supports environmental sustainability by reducing waste and replacing synthetic additives. Pomelo (*Citrus grandis*) is one of the largest citrus fruits, popular for its sweet, juicy segments and robust aroma. Its thick peel, which constitutes about 30 % of the fruit weight, is a significant source of agricultural waste. The citrus peel consists of two distinct layers, the outer pigmented layer, known as the flavedo, and the inner white layer, called albedo. Both the flavedo and albedo are rich in soluble and insoluble fibers, phenolic compounds, lipids, and organic acids (Babaoğlu et al., 2022). This fruit and its processed derivatives generate substantial waste or byproducts during consumption or processing, including peels, seeds, and pulp, which collectively constitute nearly 50 % of the fruit's initial weight (Singh et al., 2024; Tocmo et al., 2020). Beyond consumption, pomelo peel is rich in bioactive compounds such as flavonoids, essential oils, and polysaccharides, offering significant potential for use in food, cosmetic, and pharmaceutical industries (Al-Juhaimi et al., 2021). Hence, this study focuses on the formulation and evaluation of a waste extract active edible coating by encapsulating pomelo peel extract (PPE) into the nanoemulsion. It is hypothesized that incorporating PPE into the nanoemulsion matrix will enhance the antimicrobial efficacy of the coating, thereby contributing to the extended shelf life of Indian cottage cheese (ICC)/ paneer, by inhibiting microbial growth and improving quality retention of ICC/ paneer during refrigerated storage.

## 2 Material and methods

### 2.1 Materials and instruments

#### 2.1.1 Plant material

Fully matured pomelo fruits were collected from the District Solan, Himachal Pradesh, India. The peels were manually separated and were subjected to drying in a cabinet air dryer maintained at a temperature range of 40–45 °C till the moisture was reduced (<10 %). The dried peels were finely ground into powder, then sealed and stored in airtight polyethylene zip bags.

#### 2.1.2 Chemicals and reagents

Xanthan gum (mol wt.: 4.17 million Daltons, purity ≥99 %, viscosity 1400 cP at 1 % *w*/*v*, 25 °C), Tween 80, methanol, HPLC (High-Performance Liquid Chromatography) grade, sodium carbonate, aluminum chloride, sodium nitrite rutin (RU), sodium hydroxide, 1,1-diphenyl-2-picrylhydrazyl (DPPH), Folin–Ciocalteu reagent, and gallic acid were purchased from Loba Chemical Private Limited. Culture media, including nutrient agar, nutrient broth, potato dextrose broth, potato dextrose agar, ampicillin antibiotic, fluconazole antibiotic, and Muller Hinton agar (MHA), were purchased from Himedia.

#### 2.1.3 Microbial cultures

Standard test bacterial cultures, i.e., *Bacillus cereus* (MTCC 430)*, Shigella boydii* (MTCC 11947)*, Enterococcus faecalis* (MTCC 430)*, Escherichia coli* (MTCC 1687)*, Vibrio cholerae* (MTCC 3906)*, Pseudomonas aeruginosa* (MTCC 1688), and fungal culture, i.e., *Candida albicans* (MTCC 227)*, Fusarium oxysporum* (MTCC 9668)*, Rhizopus stolonifer* (MTCC 958) were procured from IMTECH, Chandigarh.

#### 2.1.4 Instruments

The instruments utilized in this research included a spectrophotometer (Thermo Fisher Scientific, Model Evolution 201), magnetic stirrer (Laby Instrument Industry, Model MS-2), centrifuge (Thermo Scientific), digital balance (Citizon, Model Y220), digital refractometer (Erma Inc.), digital pH meter (Systonic, Model S-906), texture analyzer (TA XT Plus, Stable Micro Systems), probe sonicator (Cole Parmer Ultrasonic Processor, CPX130), zeta sizer (Malvern Instruments Ltd., Malvern, WR14 1XZ, UK), and a field emission scanning electron microscope (FESEM) (Nova Nano SEM 450, FEI Company, Hillsboro).

### 2.2 Extraction of pomelo peel extract (PPE)

Bioactive compounds were extracted from the powdered peel using various solvents, including methanol, hexane, and dichloromethane. A 20 g sample of the powdered peel was extracted through the Soxhlet method with 150 mL of solvent maintained at its respective boiling point, i.e., methanol (64.7 °C), hexane (68.7 °C), and dichloromethane (39.6 °C), until the thimble showed no coloration, signifying the completion of extraction. The resulting extract was concentrated by evaporating the solvent under reduced pressure using a shaker incubator set at 45 °C. The concentrated extracts were then stored in 20 mL glass vials at 5 °C for subsequent use (Chaqroune & Taleb, 2022).

### 2.3 Bioactive compounds and antioxidant analysis of pomelo peel extract (PPE)

The bioactive profile of the PPE was assessed through determination of total phenolic content (TPC), total flavonoid content (TFC), and antioxidant activity (DPPH radical scavenging assay). The total phenolic content (TPC) of the PPE was determined using the Folin–Ciocalteu method in which 1 mL of the extract was mixed with 9 mL of distilled water and 5 mL of 10 % Folin–Ciocalteu reagent followed by incubation of 5 min, 5 mL of 7.5 % sodium carbonate solution was added. The mixture was further incubated for 90 min, and the absorbance was recorded at 750 nm using a UV–Vis spectrophotometer and the results were expressed as milligrams of gallic acid equivalents per gram of dry extract (mg GAE/g), based on a standard calibration curve. However, the total flavonoid content (TFC) was estimated using the aluminum chloride colorimetric method in which 1 mL of the extract was combined with 0.3 mL of 5 % sodium nitrite and incubated for 5 min, followed by the addition of 0.3 mL of 10 % aluminum chloride. Further, 2 mL of 1 M sodium hydroxide was added, and the final volume was adjusted to 5 mL with distilled water and was measured at 540 nm in which results was expressed as milligrams of rutin equivalents per gram of dry extract (mg RU/g). In addition to that antioxidant activity was estimated by DPPH radical scavenging assay in which 1 mL of extract was mixed with 2 mL of DPPH solution followed by incubation for 30 min at absorbance 517 nm was measured and to calculate % DPPH activity by a standard formula (Aayush et al., 2024). Further, the best optimised solvent extract based on these characterizations was selected for its antimicrobial efficacy.

### 2.4 Antimicrobial activity

#### 2.4.1 Preparation of inoculum

The antimicrobial activity of waste extracts was evaluated against Gram-positive bacteria [*B. cereus* MTCC 430 and *E. faecalis* MTCC 439], Gram-negative bacteria [*V. cholera* MTCC 3904, *S. boydii* MTCC 11947, *E. coli* MTCC 1687, *P. aeruginosa* MTCC 1688, as well as three pathogenic microscopic filamentous fungi [*C. albicans* MTCC 98001, *R. stolonifer* MTCC 2591, *F. oxysporum* MTCC 284]. The Gram-positive and Gram-negative bacteria were pre-cultured in nutrient broth (NB) overnight in a rotary shaker at 37 °C. Afterward, each strain was adjusted at a concentration of 10^8^ cells/ml using 0.5 McFarland standard. The 0.5 McFarland standard is typically made by dissolving 0.5 g of barium chloride in 100 mL of distilled water along with 0.05 mL of 1.6 N sulfuric acid, creating a solution with a turbidity equivalent to an optical density of 0.08 to 0.1 at 600 nm, corresponding to approximately 1 × 10^8^ CFU/mL. The fungal inoculum was prepared from the 48 h culture of fungal isolates in Potato Dextrose Broth (PDB). The spectrophotometer was used to adjust the fungal spore density to a final concentration of 10^6^ spores/mL (Aayush et al., 2024).

#### 2.4.2 Antibacterial and antifungal activity of PPE

Antimicrobial activity of PPE was assessed using the agar well diffusion method, described by Nigussie et al. (2021) for bacterial strains and Alyousef (2021) for fungal cultures with slight modifications. The turbidity of bacterial suspension was adjusted to 0.5 McFarland standards (approximately 10^8^ CFU/mL), and fungal cultures were standardized to 1.0 × 10^6^ spores/mL. Mueller-Hinton Agar (MHA) plates were swabbed with 100 μL of bacterial suspension, while 500 μL of fungal spore suspensions were mixed with 20 mL of potato dextrose agar (PDA) for fungal cultures and allowed to solidify. Sterile wells (6 mm diameter) were prepared using a cork-borer and filled with 100 μL of the PPE sample. Ampicillin (1 mg/mL) was used as the positive control for bacterial cultures, while fluconazole (1 mg/mL) was used for fungal cultures, along with extract and distilled water as negative. Ampicillin and fluconazole were employed as positive controls for antibacterial and antifungal assays, respectively, due to their broad-spectrum efficacy against bacterial and fungal pathogens. These are reference standards for evaluating the comparative antimicrobial potential of the pomelo peel extract. At room temperature, the plates were left for 30 min to allow diffusion, followed by incubation at 37 °C for 18–24 h for bacteria and at 27 ± 2 °C for 36 h for fungi. After incubation, the antimicrobial and antifungal activities were determined by measuring the diameter in millimeters (mm) of the clear inhibition zones in plates.

### 2.5 Nanoemulsion (NE) preparation and characterization

#### 2.5.1 Optimization and preparation of pomelo Peel extract (PPE) based nanoemulsion (NE)

The method of preparing PP-NE was adapted from Aayush et al. (2024) with slight modifications in conditions. Initially, XG was prepared at varied concentrations of 0.1 %, 0.2 %, and 0.3 % (*w*/*v*) in distilled water and homogenized using continuous stirring at 1200 rpm for 150 min at 70 °C. Tween 80, serving as the emulsifier, was subsequently incorporated into the solution at varying concentrations (1.0 %, 1.5 %, 2.0 %, 2.5 %, 3.0 %, 3.5 %, 4 % w/v) and mixed for 15 min at 25 °C for aqueous phase formation.

Oil phase was prepared by dissolving PPE in concentrations ranging from 0.5 % to 5 % (w/v) in vegetable oil, i.e. soybean oil (0.5 %). The aqueous phase (XG and Tween 80) and the oil phase were then combined, and the volume was adjusted to 100 mL using DW (distilled water). The mixture underwent preliminary homogenization using a magnetic stirrer at 1200 rpm for 1 h. Subsequently, the emulsion was subjected to probe sonication at 40 kHz for 20 min, conducted in intervals of 5 min to avoid overheating. The formulated NE was stored in glass vials under different storage conditions, i.e., ambient temperature conditions (25 ± 2 °C) and refrigerated conditions (4 ± 2 °C) for further analysis.

#### 2.5.2 Characterization of nanoemulsion (NE)

##### 2.5.2.1 Stability studies of Nanoemulsion (NE)

NEs were prepared using different concentrations of biopolymer (XG), PPE, and Tween 80 and assessed for their physical stability. Samples were placed in glass containers and stored for 30 days at room temperature (25 ± 2 °C) and refrigerated temperature (4 ± 2 °C). The physical stability of the samples was monitored by observing phase separation and creaming as phase separation and creaming are indicators of physical instability in nanoemulsions, often resulting from droplet aggregation or density differences between phases (Lewińska et al., 2021).

##### 2.5.2.2 Dynamic light scattering (DLS) and polydispersity index (PDI)

The average droplet size and polydispersity index (PDI) of the prepared nanoemulsions were measured 24 h post-preparation using Dynamic Light Scattering (DLS) on a Zetasizer Nano ZS (Malvern Instruments, UK) at a fixed detection angle of 90°. To minimize multiple scattering effects and ensure an appropriate droplet concentration (∼0.005 % *w*/w), the samples were diluted to a 10^−3^ concentration. The diluted samples were evaluated and the results were recorded at 25 ± 0.1 °C with values below 0.3 indicating a narrow and uniform size distribution.

##### 2.5.2.3 Zeta potential of nanoemulsion (NE)

Surface charge of the NE particles was evaluated using a particle electrophoresis technique. A 1 mL sample of NE was dissolved in distilled water before analysis. The estimation was performed using a Zeta sizer Nano ZS Particle Sizer at 25 °C temperature.

##### 2.5.2.4 Surface morphology of a dried nanoemulsion (NE)

Morphology and structural characteristics of the nanoemulsions (NEs) was analyzed using field emission scanning electron microscopy (FE-SEM). Freeze-dried samples were affixed to aluminum stubs and coated with a thin layer of gold using a sputter coater under vacuum conditions. The observations were conducted at an accelerating voltage of 25 kV. Digital micrographs were taken at magnifications of 150× and 150,000× to visualize the detailed structure of the freeze dried NEs.

### 2.6 Antimicrobial efficacy of nanoemulsions (NE)

Different concentrations of PPE (0.5–5.0 %) based NE were assessed for antimicrobial activity by the agar well diffusion against tested strains with slight modifications (Alyousef, 2021; Nigussie et al., 2021). The preparation of bacterial and fungal cultures, inoculation on respective agar plates (MHA for bacteria and PDA for fungus), and the use of positive (ampicillin for bacteria, fluconazole for fungi) negative (distilled water), and control followed the same procedure as described for the extract. Plates were incubated at 37 °C for 18–24 h for bacteria and at 27 ± 2 °C for 36 h for fungi. The zones of inhibition were measured in millimeters (mm) after incubation.

### 2.7 Effect of PP-NE on physicochemical and microbiological attributes of ICC/ paneer

#### 2.7.1 Preparation of ICC/ paneer

The standardized milk having 4.5 % fat and 8.5 % milk solids-not-fat (MSNF) was placed in a sanitized vessel and heated directly over a flame to 90 °C for 5 min, then cooled to 80 °C. Coagulation was induced by gradually adding a 1 % citric acid solution to the milk at 75 °C with gentle stirring until curd formation occurred and the whey separated clearly. At this stage, the pH of the whey was 5.4 to 5.6. The coagulum allowed to settle for 5 min before draining the whey through muslin cloth, maintaining the whey temperature above 70 °C. The curd was collected and placed into a rectangular stainless-steel hoop (dimensions: 15 × 10 × 9 cm^3^) lined with muslin cloth. The coagulum was pressed under a pressure of 2–3 kg/cm^2^ for 15–20 min. The resulting paneer block was then taken from the hoop and immersed in pasteurized chilled water (3–5 °C) for 2 h. After immersion, the paneer blocks were transferred to a clean dish to allow excess water to drain for 10 min. The drained paneer blocks were then weighed, and representative samples were stored for further analysis (Qureshi et al., 2023).

#### 2.7.2 Application of nanoemulsion (NE) on ICC/ paneer

Fresh ICC/paneer cubes were separated into six groups. Each cube was then dipped in 500 mL of coating solution for 180 s and left to air-dry at room temperature (25 ± 2 °C) with a relative humidity of 50–60 %, placed on a tray. Uncoated samples were used as the control. The control and coated paneer samples were stored at 4 °C for 15 days. Physiological changes such as weight loss, pH, FFA value, titrable acidity, microbial analysis, and sensorial characteristics were measured, along with photographs to monitor visual changes. The quality of samples were assessed every three days.

### 2.8 Physicochemical characteristics of Indian cottage cheese (ICC)/ paneer

#### 2.8.1 Weight loss and pH

The weight loss of the coated ICC/ paneer was measured using an analytical weighing balance. The weight loss percentage of the coated and control ICC/ paneer samples stored at refrigerated conditions (4–7 °C). It was calculated by comparing the weight loss rate on each storage day. However, for pH, a digital pH meter was used for pH levels of coated and control ICC/ paneer samples (Aayush et al., 2024).

#### 2.8.2 FFA value

FFA (Free Fatty Acid) content was evaluated following the standard titrimetric procedure (AOCS, 2017). A sample of coated and control ICC/ paneer weighing between 5 and 10 g were accurately measured and dissolved in 50 mL of 95 % ethanol. The solution was heated at 65 °C with continuous stirring. These extracts were further utilized for titration using a standardized 0.1 N sodium hydroxide solution, with the endpoint indicated by the appearance of a light pink color that persisted for at least 30 s. The FFA content of coated and control ICC/ paneer was calculated and reported as a percentage of oleic acid.

#### 2.8.3 Titrable acidity

The titratable acidity (TA) of ICC/ paneer was determined using the method described by AOAC (1984). 10 g of coated and control ICC/ paneer were homogenized into a smooth paste using distilled water. The mixture was diluted to a final volume of 100 mL, followed by thoroughly mixing, and filtered through Whatman No. 2 filter paper to obtain a clear extract. The resulting filtrate was titrated with a 0.1 N sodium hydroxide (NaOH) solution, using 1 % phenolphthalein indicator. The appearance of a pink color signified the titration endpoint. Titratable acidity was expressed as a percentage of lactic acid by weight, using the standard formula.

#### 2.8.4 Microbiological analysis

Microbial analysis, including total bacterial plate counts and total yeast and mold counts, was performed following standard procedures (APHA, 1992). A 2 g samples of coated and control ICC/ paneer were placed in a sterile plastic bag and homogenized with 0.9 % saline solution, with analyses conducted at three-day intervals throughout the storage period. 10^−1^, 10^−2^, and 10^−3^ Serial dilutions were prepared, and 1 mL from each dilution was inoculated into sterile petri plates. Nutrient agar was utilized for assessing total bacterial counts, while potato dextrose agar was employed for yeast and mold enumeration. After the media solidified, the plates were incubated at 37 °C for bacterial growth and at 30 °C for yeast and mold growth. Microbial counts were recorded and expressed in logarithmic terms as Log_10_ CFU/g (Kumari & Farid, 2020).

#### 2.8.5 Sensorial characteristics

During the storage periods on days 0, 3, 6, 9, 12, 15, a semi-trained panel of 15 members assessed the samples for appearance, texture, color, flavor, and overall acceptability. Only samples that did not show visual or olfactory signs of spoilage were subjected to sensory evaluation. The evaluations were conducted using a 9-point hedonic scale, where 1 represented “dislike extremely,” 2 “dislike,” 3 “dislike moderately,” 4 “dislike slightly,” 5 “neither like nor dislike,” 6 “like slightly,” 7 “like,” 8 “like very much,” and 9 “like extremely. The 9-point hedonic scale was used to evaluate the likability of sensory attributes by numerical ratings, enabling an objective analysis of overall acceptability (Miranda et al., 2022).

### 2.9 Statistical analysis

Each experiment, excluding DLS and FESEM, was conducted in triplicate, and the mean ± standard deviation (SD) is used to report all data. One-way analysis of variance (ANOVA) was used for statistical analysis of the data from GraphPad Prism 5.02 software. *P* value ≤0.05 were regarded as statistically significant.

## 3 Results and discussions

### 3.1 Characterization of pomelo peel extract (PPE)

#### 3.1.1 Physicochemical analysis of pomelo Peel extract (PPE)

**Fig. 1:**
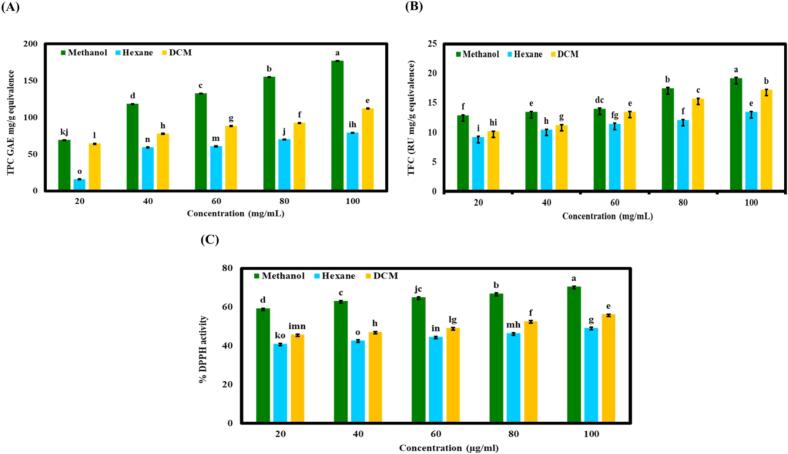
Physiochemical analysis of PPE extracted using methanol, hexane, and dichloromethane as solvents: (A) Total phenolic content (TPC), expressed as mg gallic acid equivalents (GAE)/g dry extract (20–100 mg/mL); (B) Total flavonoid content (TFC) expressed as mg rutin equivalents (RU)/g dry extract (20–100 mg/mL); (C) DPPH radical scavenging activity (%) at 20–100 μg/mL. Different letters (a-o) indicate significant difference (*p* < 0.05) between the solvents at the same concentration. Data are presented as mean ± standard deviation (*n* = 3).

Extracts utilizing the Soxhlet extraction technique with different solvents through the Folin-Ciocalteu reagent assay. A comparative evaluation of methanol, hexane, and dichloromethane indicated significant differences in their efficacy for measuring antioxidant activity, TPC, and TFC. Methanol consistently demonstrated superior performance across all parameters at highest concentration achieving the highest % DPPH activity (70.49 ± 0.23), followed by dichloromethane (56.19 ± 0.14) and hexane (49.28 ± 0.28) at 100 μg/mL. Additionally, methanol extracts exhibited the highest TPC (177.06 ± 0.08 GAE mg/g equivalence), while dichloromethane and hexane yielded TPC values of 112.41 ± 0.33 GAE mg/g equivalence and 79.52 ± 0.21 GAE mg/g equivalence, respectively. Similarly, in TFC methanol showed the greatest extraction efficiency (19.20 ± 0.12 RU/g equivalence), followed by dichloromethane (17.23 ± 0.09 RU/g equivalence) and hexane (13.46 ± 0.09 RU/g equivalence). Hence, methanol is the most effective solvent for extracting antioxidants, phenolics, and flavonoids, followed by dichloromethane whereas hexane was found to be the least effective extraction efficiency. Fig. 1 demonstrates that the concentration is directly related to the levels of phenolics, flavonoids, and antioxidant activity. As the concentration decreases, there is a corresponding decline in these values. Hence, that is why the methanolic extracts were used for incorporation into the nanoemulsion edible coating. Similar studies observed by Al-Juhaimi et al. (2021) that methanol is the most effective solvent in extracting various bioactive compounds. Another study reported by Papoutsis et al. (2018) found that absolute methanol and 50 % acetone resulted in the highest total phenolic content (TPC) yields from lemon pomace waste.

#### 3.1.2 Antimicrobial activity of pomelo peel extract (PPE)

**Fig. 2:**
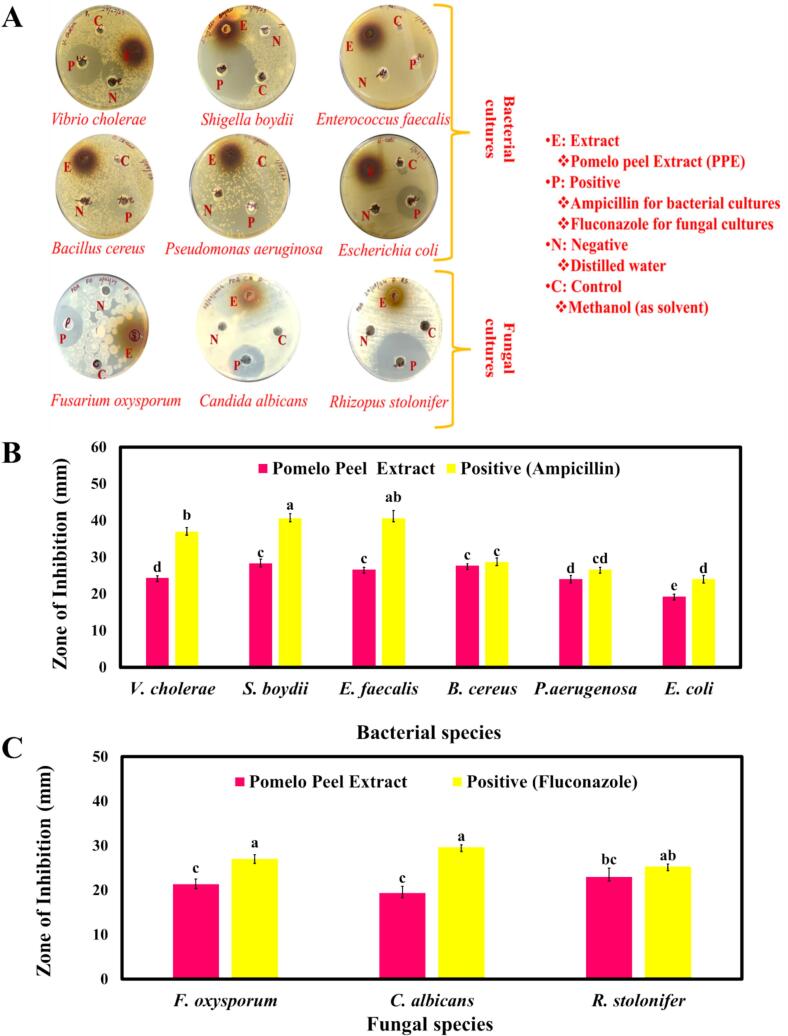
Antibacterial and antifungal activity of PPE. (A) Representative agar diffusion plates showing zones of inhibition against six bacterial strains: *P. aeruginosa, E. faecalis, E. coli, V. cholerae, B. cereus, S. boydii.* and three fungal strains: *F. oxysporum*, *C. albicans*, *R. stolonifer* (B) Quantitative comparison of antibacterial activity between PPE and the positive control. (C) Quantitative comparison of antifungal activity between PPE and the positive control. Different letters (a-d) indicate statistically significant differences (*p* < 0.05) between the extract and positive control for the same pathogen. Data are presented as mean ± standard deviation (*n* = 3).

The antibacterial activity of PPE was evaluated against six bacterial strains, namely, *V. cholerae, S. boydii, E. faecalis, B. cereus, P. aeruginosa,* and *E. coli*. The inhibition zones (mm) measured with standard deviations showed varying antibacterial efficacy as shown in Fig. 2(A), the inhibition zones for *V. cholerae* were 24.3 ± 0.57 mm, while *S. boydii* exhibited the highest inhibition zone of 28.3 ± 1.1 mm. However, *E. faecalis* showed an inhibition zone at 26.6 ± 0.57 mm and *B. cereus* showed at 27.6 ± 0.5 mm. Furthermore, *P. aeruginosa* (24 ± 1 mm) and *E. coli* (19.3 ± 0.57 mm), showed minimum inhibition zones as compared to other tested bacterial strains which is summarised in Fig. 2(B). The results indicated the stronger antibacterial properties of pomelo peel as it inhibits bacterial growth through mechanisms such as disrupting microbial respiration and increasing plasma membrane permeability, leading to bacterial cell death through ion leakage (Ajayi et al., 2019, Górniak, Bartoszewski, & Króliczewski, 2019). Natural antibacterial agents, particularly plant extracts, contain hydrophobic compounds such as essential oils and polyphenols, which can penetrate the thick but permeable peptidoglycan layer of Gram-positive bacteria more easily than the complex external membrane of Gram-negative bacteria (Nourbakhsh, Lotfalizadeh, Badpeyma, Shakeri, & Soheili, 2022). Consequently, PPE exhibited greater antibacterial effects against both Gram-positive and Gram-negative bacteria. Furthermore, the antifungal activity of PPE was assessed against three common food-spoilage fungi, i.e., *F. oxysporum, C. albicans,* and *R. stolonifer* (Fig. 2A). The results revealed that PPE exhibited an inhibition zone of 19.33 ± 1.52 mm against *C. albicans* which was the lowest inhibition zone observed but 21.33 ± 1.15 mm against *F. oxysporum*. However, for *R. stolonifer*, PPE showed the highest inhibition (23 ± 2 mm) among the other tested fungi which is summarised in Fig. 2(C). This strong antifungal activity is attributed to potent bioactive compounds such as limonoids, which disrupt multiple cellular processes and interfere with key metabolic pathways (Dubey et al., 2019). The variation in antifungal effectiveness is due to the distinct profile and concentration of bioactive compounds in PPE, which impact cell wall synthesis, membrane integrity, protein and enzyme function, and oxidative stress in fungal cultures (Patrignani, Siroli, Serrazanetti, Gardini, & Lanciotti, 2015). This antifungal effect can be attributed to the disruption of ergosterol synthesis, a critical biochemical pathway that affects fungal cell metabolic activities, membrane permeability, and mitochondrial function (Eliaš, Tóth Hervay, & Gbelská, 2024, Guo, Liang, Xiao, & Ge, 2023). The antimicrobial activity of PPE is directly related to their polyphenol content, with higher polyphenol levels contributing to stronger antimicrobial effects (Nassarawa et al., 2024).

### 3.2 Stability studies of nanoemulsion (NE)

The stability of NE formulations incorporating XG, Tween 80, and PPE as the oil phase was assessed under varying concentrations and storage conditions. XG concentrations of 0.1 %, 0.2 %, and 0.3 % were combined with Tween 80 at different levels (1 %, 1.5 %, 2 %, 2.5 %, 3 %, 3.5 %, 4 %) while PPE concentrations varied from 0.5 % to 5.0 %. The formulations were subjected to stability tests under refrigerated (4 °C), room temperature (30 °C), and elevated temperature (60 °C) conditions. Additionally, centrifugation at 1200 rpm was conducted to evaluate phase separation (PS), stability (S), and instability (US). Formulations with 0.1 % XG (Fig. S1A.) and Tween 80 concentrations (1 % and 2 %) displayed phase separation across all storage conditions and centrifugation tests, indicating poor stability but at 1.5 % of Tween 80 concentration found a highly stable formulation, with no phase separation at 4 °C and 30 °C, while instability persisted at 60 °C. Furthermore, at 0.2 % (Fig. S1A.) of XG concentration with 1.5 % Tween 80 exhibited consistent stability across all conditions, including centrifugation. This suggests that an optimal surfactant-to-polymer ratio is crucial for stabilizing nanoemulsions. However, increasing the XG concentration to 0.3 % (Fig. S1A.) showed instability as clumps were formed. This might be due to excessive viscosity impeding uniform droplet distribution. The most stable formulation was achieved using 0.2 % XG, and 1.5 % Tween 80, demonstrating resilience across varying temperatures and centrifugation. Phase separation in formulations due to low surfactant-to-polymer ratios as the surfactant concentration was too low to decrease the interfacial tension significantly to produce and maintain a stable emulsion (Abbasi Moud, 2022). Several formulations remained stable at varied temperatures but were unstable during centrifugation, as the mechanical force was applied at increased rotational speeds and these forces created significant differences in density-driven acceleration between the oil and water phases, which caused strong interfacial and shear stresses on the droplet interfacial layer (Griffith & Daigle, 2020). Furthermore, XG also increases the aqueous phase's viscosity, which restricts the mobility and the collisions of droplets. Hence, this viscoelastic matrix helps to prevent flocculation and coalescence by providing steric stabilization around the droplets (Lewińska et al., 2021; Ribeiro et al., 2024). Moreover, the bioactive content found in PPE is rich in flavonoids and essential oils, which exhibit amphiphilic properties that might be useful in enhancing stability by the reinforcement of the interfacial film (Gao et al., 2023). These bioactive compounds interacted with Tween 80 molecules at the interface, thus making the surfactant layer more stable and enhancing its ability to bear stress. However, the antioxidant property of the extract may protect the system against oxidative degradation, which could destabilize the emulsion (Said et al., 2023). Li et al. (2022) found that XG enhanced stability, viscosity, and flow behavior when combined with Tween 80 in rice bran oil-based emulsions. Similarly, curcumin-loaded nanoemulsions stabilized with Tween 80 demonstrated a smaller particle size with enhanced physical and storage stability (Aayush et al., 2024, Aayush et al., 2025). Therefore, at 1.5 % Tween 80 and 0.2 % xanthan gum showed stability for more than 30 days under ambient conditions, while at refrigerated conditions, the nanoemulsion was stable for 60 days. This indicated that the 0.2 % xanthan gum with 1.5 % Tween 80 improved the stability of the formulations for extended durations.

### 3.3 Antimicrobial activity of PP-NE

**Fig. 3:**
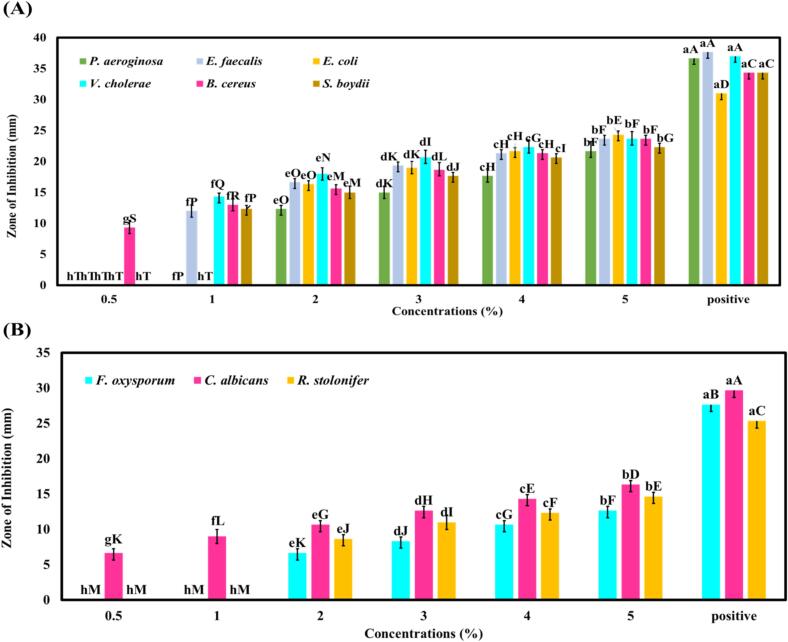
Antibacterial and antifungal activity of the PP-NE against six bacterial strains: *P. aeruginosa, E. faecalis, E. coli, V. cholerae, B. cereus, S. boydii,* and three fungal strains: *F. oxysporum*, *C. albicans*, *R. stolonifer* (A) Graphical representation of antibacterial activity at different nanoemulsion concentrations (0.5–5 %) compared with positive control (ampicillin). (B) Graphical representation of antifungal activity at different nanoemulsion concentrations (0.5–5 %) compared with positive control (fluconazole). Different letters (a-h) indicate statistically significant differences (*p* < 0.05) among the nanoemulsion concentrations and positive control within the same pathogen. Different letters (A-T) indicate statistically significant differences (p < 0.05) among the different pathogens with nanoemulsions concentrations and positive control. Data are presented as mean ± standard deviation from three independent experiments (*n* = 3).

The in vitro antimicrobial efficacy of the PPE-loaded NE was assessed against six bacterial cultures (*E. faecalis, E. coli, V. cholerae, B. cereus, S. boydii*, and *P. aeruginosa*) and three fungal cultures (*R. stolonifer, F. oxysporum, and C. albicans* at various concentrations (0.5 %, 1 %, 2 %, 3 %, 4 %, and 5 %), using ampicillin and fluconazole as positive controls for bacterial and fungal strains, respectively and all the concentrations demonstrated notable antibacterial activity against the tested pathogens (Fig. 3A). The control NE, formulated without PPE in the XG solution, showed no inhibition zones. The antibacterial activity of the PP-NE was concentration-dependent, with larger inhibition zones observed at higher PPE concentrations (5 %). At 2 % PP-NE, effective inhibition zones were detected against all tested bacterial strains. However, at the lowest concentration i.e., 0.5 % of PP-NE, a measurable zone of inhibition (9.3 ± 1.15 mm) was observed only against *B. cereus* and 6.66 ± 0.57 zone in case of *C. albicans* and at 1 % PP-NE, inhibition zones were observed for all the tested strains, but no zones of inhibition were detected against *E. coli* and *P. aeruginosa*. However, at a 2 % PP-NE concentration, all bacterial strains showed inhibition zones measuring 16.67 ± 0.57 mm (*E. faecalis*), 16.33 ± 0.57 mm (*E. coli*), 18 ± 1 mm (*V. cholerae*), 15.67 ± 0.57 mm (*B. cereus*), 15 ± 1 mm (*S. boydii*), and 12.33 ± 0.57 mm (*P. aeruginosa*). In the case of fungal cultures, the PP-NE demonstrated zones of inhibition of 7 ± 1 mm, 5.3 ± 0.57 mm, and 4.3 ± 0.57 mm for *R. stolonifer*, *F. oxysporum*, and *C. albicans*, respectively, at the minimum inhibitory concentration of 2 % (Fig. 3B). A higher concentration of PPE led to enhanced antimicrobial efficacy against both bacterial and fungal pathogens. In contrast, the control sample, i.e., without PPE, showed no inhibition zones. The differences in the zone of inhibition against tested microorganisms that responded to the PP-NE may be due to variations in their physical properties, such as the structure and composition of their cell walls, and their natural sensitivity to the active compounds in PPE. Hence, it can be concluded that the potential of PP-NE as an effective antimicrobial agent, with its activity improved as the concentration increases. A similar study was reported by Meydanju et al. (2022) that lemon peel powder and xanthan gum, enhanced with TiO_2_ and Ag nanoparticles-based film exhibits strong antimicrobial properties against *E. coli* and *S. aureus.* The mechanism behind PP-NE might due to the synergistic effects of bioactive compounds in pomelo peel, such as flavonoids (including hesperidin), essential oils, and organic acids. These compounds solubility and stability are enhanced by the NE, amplifying their antimicrobial properties (Aayush et al., 2024). These components can damage the integrity of the microbial cell membrane, which results in the loss of vital nutrients essential for microbial growth and, ultimately, cell death (El-Sherbiny et al., 2024). The extract's overall efficacy can also be increased by the nanoscale emulsion's ability to prolong contact with the bacteria and release antibiotic chemicals over time. The nanoparticles have a greater capacity to interact with the microbial cell surfaces because they penetrate the cell wall more effectively than bulk extracts (Koch et al., 2024). The findings of this study indicate that PP-NE encapsulates bioactive compounds from PPE, effectively inhibiting bacterial and fungal pathogens at a 2 % PPE concentration. This 2 % PPE concentration demonstrated inhibitory effects against all tested pathogens, making it suitable for further test analysis and subsequent application on ICC/ paneer.

### 3.4 Dynamic light scattering (DLS), polydispersity index (PDI), and zeta potential of stable Nanoemulsion (NE)

The optimised NE based coating, prepared with a combination of 0.2 % of XG, 1.5 % of Tween 80, and 2 % of PPE, was characterized for their droplet size, PDI and zeta potential. The results revealed its stability and potential effectiveness (Fig. S2). The droplet size of 287.7 nm (Fig. S2A) polydispersity index (PDI) of 0.116. These results suggest that nanometric range, enhancing the coating's functional properties, such as improved surface coverage and penetration (Joshi & Bhattacharyya, 2011) and the narrow size distribution of the NE droplets, indicating uniformity and stability of the system. A low PDI value (< 0.3) is desirable as it implies better long-term stability and consistent performance of the coating (Sneha & Kumar, 2022). The zeta potential value of −30 mV (Fig. S2B) is significant, as it indicates the electrostatic repulsion between droplets, which helps prevent aggregation and coalescence (Kamble et al., 2022). However, zeta potential values greater than ±30 mV are associated with good stability in colloidal systems (Midekessa et al., 2020).

### 3.5 Field emission scanning electron microscope (FESEM)

**Fig. 4:**
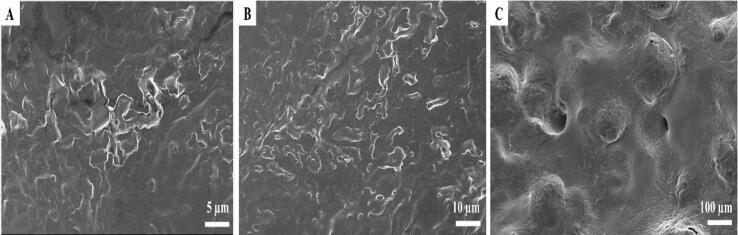
Field emission scanning electron microscopy (FE-SEM) images of freeze-dried pomelo peel nanoemulsion (PP-NE): (A) microstructure at 2000× magnification with a scale bar of 5 μm; (B) at 500× magnification with a scale bar of 10 μm; (C) at 100× magnification with a scale bar of 100 μm.

The FESEM images of the PP-NE were analyzed at three different magnifications, which revealed distinct morphological characteristics across varying magnifications. At higher magnification (Fig. 4A), the surface appeared relatively compact with slight irregularities and wrinkles, indicating the successful formation of nano-sized droplets. The presence of crevices and folds suggested the encapsulation of the oil phase within the polymeric matrix. However, Fig. 4(B) indicates that the surface displayed a heterogeneous structure with visible undulations and dispersed droplets, reflecting a stable nanoemulsion network that may help resist phase separation. The surface texture observed here could be influenced by the emulsifier concentration, which plays a crucial role in stabilizing the droplets and preventing coalescence. It represents the encapsulated bioactive components of the pomelo peel, that is stabilized by surfactants and emulsifiers used in the formulation (Ozogul et al., 2022). Furthermore, at the macro level, i.e., 100 μm scale (Fig. 4C), the larger surface features are distinct pores and cavities of the nanoemulsion. These porous structures likely resulted from moisture loss during the freeze-drying process, forming voids within the matrix. However, the surface roughness observed at 100 μm might be due to the evaporation when samples were prepared for FESEM analysis by the method of freeze drying also called lyophilization. The porous structure created by lyophilized microencapsulated powders helps to preserve reconstitution properties and extends their shelf life (Ward & Matejtschuk, 2021). Additionally, the boundaries of the particles indicate effective encapsulation, likely facilitated by the interfacial properties of the emulsion's oil and aqueous phases (Ravera et al., 2021).

### 3.6 Effect of PP-NE on the shelf life of ICC/ paneer

**Fig. 5:**
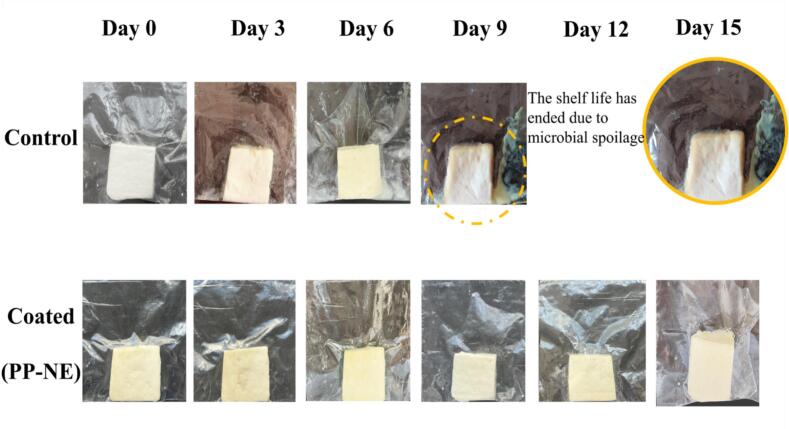
Changes in the visual appearance of ICC/ paneer during refrigerated storage.

The application of PP-NE coating was assessed for its impact on the storage quality of ICC/ paneer during 15 days. Visual observations showed that the control ICC/ paneer exhibited spoilage by the 6th day. However, all quality analyses in control samples were conducted only until the 6th day, as unpleasant odor developed by the 7th day, although visual observations, as shown in Fig. 5, were recorded until the 9th day for reference. The coated ICC/ paneer, on the other hand, showed less spoilage as compared to the control by the 15th day, which clearly explains the longevity of the coating and hence was recorded for 15 days. Additionally, key quality parameters such as weight loss, pH, free fatty acid (FFA) content, titrable acidity, total plate count, total yeast mold count, and sensory attributes were evaluated to comprehensively analyze the coating's effectiveness.

#### 3.6.1 Weight loss

**Fig. 6:**
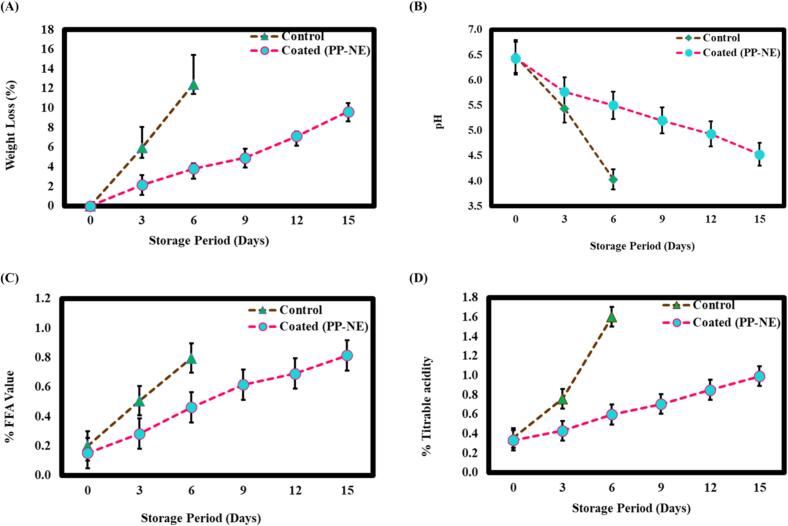
Physicochemical characterization of coated and control ICC/paneer during storage under refrigerated conditions: (A) Weight Loss; (B) pH; (C) Free fatty acid (FFA) content; (D) Titrable acidity (%), measured at 3-day intervals. Data represent mean ± standard deviation (*n* = 3).

The application of a PLE-based NE coating on ICC/ paneer demonstrated significant efficacy in mitigating weight loss over a 15-day storage period compared to control samples. The control ICC/ paneer had a significant weight loss and quick moisture loss by day 6 (12.43 ± 2.96 %), which can be shown in Fig. 6A, Loss of moisture is the indication of dehydration and deterioration (Goyal & Goyal, 2016). In contrast, the PP-NE coated samples displayed slower and more controlled weight loss (7.13 ± 0.39 %) reduction observed by day 9 and 9.62 ± 0.47 % by day 15 which can be seen in Fig. 6A. Hence, the difference in weight loss patterns can be attributed to the formation of a protective barrier by the PP-NE coating which effectively minimizes water vapor transmission and hinders microbial growth, thereby extending the shelf life of the ICC/ paneer (Jafarzadeh et al., 2021). Similar studies indicated the constant values of water activity which is accountable for the weight loss (Polat Yemiş et al., 2022). Since, water activity and weight loss are directly proportional to each other in the case of ICC/ paneer as controlled water activity effectively reduces moisture loss and enhances product stability (Arulkumar et al., 2023).

#### 3.6.2 pH

The preservation impact of PPE-based nanoemulsion (PP-NE) on pH of ICC/ paneer was monitored over a 15-day storage period under refrigerated conditions, in which coated samples showed a slight decrease in pH than the control ICC/ paneer (Fig. 6B). However, the pH of the control sample declined sharply to 4.03 ± 0.15 by day 6, indicating rapid microbial activity and spoilage. In contrast, the PP-NE-coated ICC/paneer exhibited a more gradual decline, retaining a relatively higher pH of 4.53 ± 0.20 even on day 15. This delayed acidification is likely due to the antimicrobial properties of the coating, which inhibited the growth of spoilage-causing microorganisms. The nano-sized molecules of PP-NE facilitated the formation of a uniform and protective barrier on the paneer surface, effectively reducing microbial colonization and metabolic activity (Dhanasekaran et al., 2024). This barrier effect contributed to a slower pH decline by delaying proteolysis and acid production, thereby preserving texture and sensory attributes. Similar findings were reported by Prajapati et al. (2023), who demonstrated the effectiveness of edible coatings in maintaining pH stability during storage.

#### 3.6.3 FFA value

Free fatty acids (FFA) value is the amount of lipid hydrolysis which shows the release of fatty acids from triglycerides because of enzymatic breakdown or microbial lipase activity (Rodriguez-Sanchez et al., 2021). It is a crucial parameter of lipid deterioration and food product quality, especially in dairy products like ICC/ paneer. High percentage FFA values indicate advanced spoiling, which can result in rancid flavors, off-odours, and poor texture (Aman, 2024). However, this study shows that ICC/ paneer coated with PP-NE had a lower FFA value during the storage period than the control also shown in Fig. 6C. The FFA value of the coated sample increased slowly, up to 0.81 ± 0.09 % by day 15, whereas at day 6, the control showed a significant increase up to 0.80 ± 0.13 %. PP-NE coated sample showed a slow increase in FFA value because of the antibacterial properties of the NE, which reduce lipolytic activity by inhibiting the growth of bacteria that hydrolyze lipids. Similarly, (Gharibzahedi & Mohammadnabi, 2017) revealed that edible coatings containing essential oil that acts as a protective barrier reduce the lipolytic activity that is responsible for accelerating hydrolytic rancidity and increase FFA value.

#### 3.6.4 Titrable acidity

Titrable acidity is a key indicator of the quality of perishable commodities like ICC/ paneer (Kaur et al., 2025). It reflects the metabolic activity of lactic acid bacteria (LAB), which ferment residual lactose into lactic acid and other organic acids, which can cause microbial spoilage, enzymatic activity, and biochemical changes that can negatively impact the taste, texture, and shelf life of the cheese (Raghuvanshi et al., 2025). This accumulation of acids results in a higher concentration of hydrogen ions, which increases titratable acidity. Additionally, spoilage microorganisms may contribute to further acid production during extended storage periods (Ibrahim et al., 2021). In the present study (Fig. 6D), the coated ICC/paneer with PP-NE demonstrated significantly lower titratable acidity levels compared to the control samples during refrigerated storage. The titratable acidity values of control ICC/paneer (0.36 ± 0.04 %) and the PP-NE coated samples (0.33 ± 0.06 %) were almost the same initially. Furthermore, by day 6, it was observed that the control showed 1.60 ± 0.03 % by day 6, while the PP-NE coated sample showed a lower value of 0.63 ± 0.05 %. However, by day 15, the PP-NE coated ICC/paneer effectively delayed spoilage progression (0.99 ± 0.06 %) relative to the control throughout the storage period. Similarly, Mileriene et al. (2021) found that the acidity of curd cheese increased over time due to the activity of lactic acid bacteria (LAB).

#### 3.6.5 Microbial analysis

**Fig. 8:**
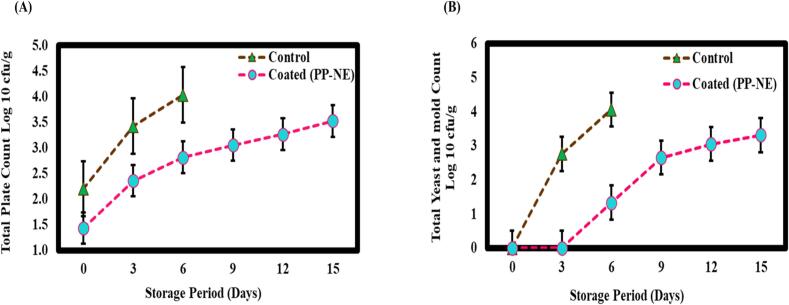
Microbial analysis of coated and control ICC/ paneer during storage under refrigerated conditions (A) Total plate count (TPC), (B) Total yeast and mold count (YMC). Data are expressed as mean ± standard deviation (*n* = 3).

Microbial analysis is essential for evaluating shelf life as the growth and activity of microorganisms significantly affect the quality and safety of food products over time. This study assesses the efficacy of a PP-NE-based coating in extending the shelf life of ICC/ paneer's by evaluating the microbial load through total plate count (TPC) and yeast mold count (YMC). TPC is used to estimate the number of viable microorganisms, including bacteria, yeasts, and molds, present at the surface of the sample. This method involves growing microorganisms from the sample on a nutrient-rich agar plate and counting the resulting colonies. TPC results for both control and coated samples were expressed in log_10_ CFU/mL over a 15-day storage period. Fig. 8A illustrates that control samples exhibited a rapid increase in microbial growth, starting from approximately 2.20 ± 0.17 log_10_ CFU/mL at day 0 and reaching about 4.03 ± 0.03 log_10_ CFU/mL by day 6. However, the PP-NE coated samples showed a significantly slower increase in microbial load, maintaining counts between approximately 2.10 ± 0.17 log_10_ CFU/mL at day 0 and 3.52 ± 0.01 log_10_ CFU/mL at day 15. This indicates that the coating effectively reduced microbial growth by approximately 1 log cycle compared to control samples over the period of 15 days. Furthermore, yeast and mold count refers to the number of yeast cells and mold spores present in a sample, naturally present in food, beverages, or other environments. YMC count in the control sample increased rapidly from day 0 to day 3 i.e., 2.75 ± 0.24 log_10_ CFU/mL, and then continued to rise steadily until day 6 (4.06 ± 0.01 log_10_ CFU/mL), that is summarised in Fig. 8B. The ICC/ paneer coated with PLE exhibited a delayed microbial growth until day 6. The results show that PP-NE is significantly more effective than the control at prolonging the microbiological shelf life of ICC/ paneer. PLE-coated ICC/ paneer showed a slow increase in microbial growth (3.31 ± 0.04 log_10_ CFU/mL) at day 15. However, at Day 6, the coated samples had 1.33 ± 1.15 log_10_ CFU/mL as compared to 4.06 ± 0.01 log_10_ CFU/mL in the control which suggested that nanoscale emulsion particles in PP-NE provide superior surface coverage and penetration into the ICC/ paneer matrix, which creates a microbial barrier and contributes to its increased antimicrobial activity. Furthermore, bioactive substances found in pomelo peel, such as flavonoids, polyphenols, and essential oils, have antimicrobial qualities by rupturing microbial cell membranes, interfering with metabolism, and inhibiting enzymes that regulate microbial development (Hou et al., 2022). Hence, the prolonged release of antimicrobial compounds from the NE retains an efficient concentration of bioactive components during the storage period, which is responsible for shelf life enhancement. Similar studies showed the efficiency of citral NE for the shelf life extension of fresh-cut pineapple (Prakash et al., 2020). This study explores a composite coating composed of whey protein isolate, XG, and clove oil, which demonstrated significant effectiveness in preserving quality attributes and prolonging the shelf life of tomatoes (Kumar & Saini, 2021). Additionally, XG-based coatings were assessed for their ability to minimize decay and sustain the quality of mangoes, emphasizing their potential as health-conscious and environmentally sustainable solutions (Prasad et al., 2022).

#### 3.6.6 Sensory analysis

**Fig. 7:**
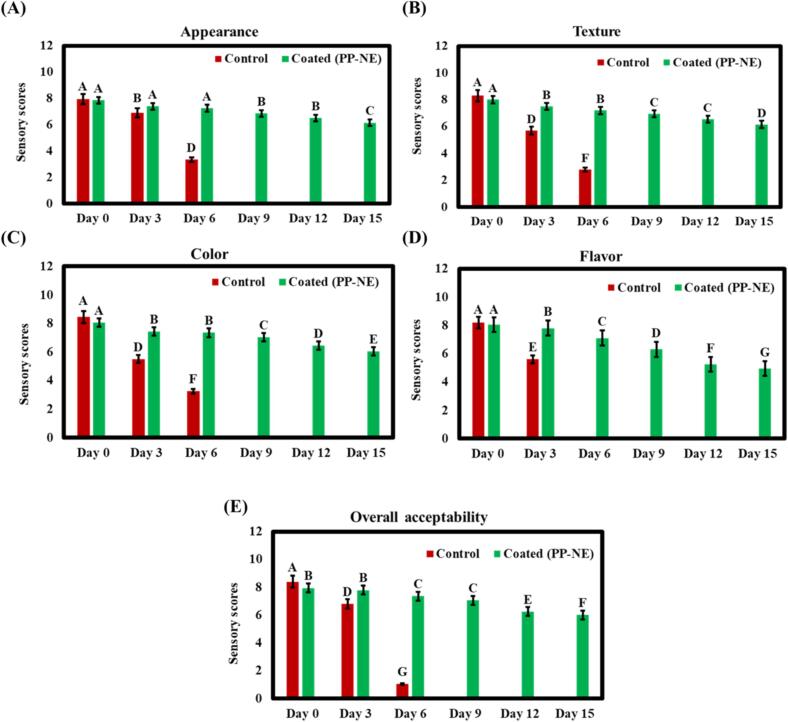
Sensory evaluation of coated and control ICC/ paneer during storage under refrigerated conditions based on appearance, flavor, color, texture, and overall acceptance. Different letters (A–G) indicate significant differences (*p* < 0.05) between different treatments across storage days. Data are expressed as mean ± standard deviation (n = 3).

The sensory qualities of ICC/ paneer, including its appearance, color, flavor, texture, and overall acceptability, are positively influenced by utilizing PP-NE over a 15-day storage period. A 9-point hedonic scale was used to compare these effects with an uncoated control sample, and the results showed significant variations between the two samples which can be shown in Fig. 7. The control sample in case of appearance showed a rapid decline in score from 7.85 ± 1.29 on day 0 to 3.4 ± 0.58 by day 6. On the other hand, the PP-NE coated ICC/ paneer retained a better score of 6.15 ± 1.29 even on day 15. Similarly, in the case of color, control showed a significant decrease in the score from day 0 (8.45 ± 0.60) to day 6 (3.25 ± 0.58). On the other hand, the coated sample retained color stability, scoring 6.05 ± 0.82 on day 15 because oxidation had a direct impact on color which caused slight discoloration. Another parameter is texture which showed significant changes in PP-NE as compared to the control sample. The control sample's texture score was 8.3 ± 0.73 on day 0 and 2.8 ± 0.83 on day 6. On the other hand, the coated ICC/ paneer showed a slow decrease, retaining a score of 6.15 ± 0.71 on day 15. The texture of ICC/ paneer appears fine on day 0, but slime formation occurs on the 6th day. Furthermore, flavor, one of the most important parameters of sensorial attributes, showed similar trends. Lipid oxidation and microbial activity produced off-flavors, causing the control sample's flavor scores to rapidly deteriorate from day 0 (8.2 ± 0.71) to day 3 (5.6 ± 0.71) while the coated sample showed a slow drop with a score of 4.95 ± 0.75 at day 15 in the case of coated ICC/ paneer. However, the control ICC/paneer showed visible discoloration, signalling microbial spoilage by the 6th day of storage, and due to the presence of fungal contamination and foul odor, no sensory scores were recorded. Lastly, overall acceptability is a sensory feature that represents the average consumer's subjective evaluation of the product's degree of likeability based on all of its sensory characteristics. Change in score observed on day 6, the acceptability of the control sample had sharply decreased to 1.06 ± 0.82, indicating compromised sensory qualities. On the other hand, the PLE-coated ICC/ paneer continued to have higher acceptance scores (6 ± 0.68) until day 15, highlighting the NE's protective and shelf-life-extending properties. The findings of the present study are like those reported in previous research (Polat Yemiş et al., 2022; Prajapati et al., 2023).

## 4 Conclusion

This study explored the utilization of PPE as a functional compound, highlighting its significant potential in preventing microbial growth on perishables. This efficacy is primarily attributed to the high concentrations of phenolic (177.06 ± 0.08 GAE mg/g equivalence) and flavonoid compounds (19.20 ± 0.12 RU/g equivalence) present in PPE, which are responsible for its antimicrobial properties (70.49 ± 0.23 % DPPH inhibition). Further, the antimicrobial efficacy of PPE was validated against both Gram-positive and Gram-negative food spoilage microorganisms. Among Gram-negative strains, *Shigella boydii* showed the highest susceptibility (28.3 ± 1.1 mm), while *Bacillus cereus*, a Gram-positive spore-forming bacterium, exhibited a notable inhibition zone (27.6 ± 0.5 mm). Additionally, significant antifungal activity was observed against *Rhizopus stolonifer* (23 ± 2 mm), indicating PPE's broad-spectrum antimicrobial potential. Hence, the bioactivity of PPE was utilized in developing nanoemulsions. The nanoemulsion (NE) formulated using 2 % PPE, 0.2 % xanthan gum (XG), and 1.5 % Tween 80 demonstrated effective stabilization under diverse conditions, including room temperature and refrigeration, with no signs of phase separation, creaming, or sedimentation for up to 60 days. Morphological characterization using Field Emission Scanning Electron Microscopy (FE-SEM) further validated the structural integrity of the PPE-NE, emphasizing its superior structure attributed to the bioactive components of PPE. The NE containing 2 % PPE exhibited significant antimicrobial activity against all tested pathogens. Among the bacterial strains, *Vibrio cholerae* showed the maximum inhibition zone of 18 ± 1 mm, followed by *Enterococcus faecalis* (16.67 ± 0.57 mm), *Escherichia coli* (16.33 ± 0.57 mm), *Bacillus cereus* (15.67 ± 0.57 mm), *Shigella boydii* (15 ± 1 mm), and *Pseudomonas aeruginosa* (12.33 ± 0.57 mm). Among the fungal strains, *Rhizopus stolonifer* showed the highest zone of inhibition (7 ± 1 mm), followed by *Fusarium oxysporum* (5.3 ± 0.57 mm) and *Candida albicans* (4.3 ± 0.57 mm) Although a 5 % PPE formulation demonstrated stronger antimicrobial activity, it led to poor mixing, phase separation, and sedimentation, which compromised uniformity and coating application. Therefore, 2 % PPE was determined to be the optimal concentration, providing a balance between bioactivity and physical stability. When applied as a coating on ICC/paneer, the PPE-NE significantly reduced weight loss by day 15, the PP-NE-coated samples exhibited significantly lower weight loss (9.62 ± 0.47 %). Microbial analysis revealed that the total plate count remained at 3.52 ± 0.01 log₁₀ CFU/mL in coated samples on day 15, which is considered an acceptable range for the consumption in ICC/ paneer. Similarly, yeast and mold counts were substantially lower in coated samples till 15 days (3.31 ± 0.04 log₁₀ CFU/mL) than in the control at the 6th day (4.06 ± 0.01 log₁₀ CFU/mL). Additionally, the PP-NE coating delayed the reduction in pH (4.53 ± 0.2), suppressed FFA accumulation (0.81 ± 0.09 %), and limited titratable acidity (0.99 ± 0.06 %). Sensory evaluation also confirmed higher acceptability scores for coated samples. Further studies focused on expanding the application of PPE-NE coatings across various types of produce, improving formulation stability, strengthening coating adhesion and durability, and ensuring uniform application. Moreover, research on the migration and interaction of nano-encapsulated bioactives with food components is essential for improving risk assessment and ensuring safe consumption. Large-scale production and packaging compatibility studies will also be critical for commercial adoption and application in the food preservation sector.

## CRediT authorship contribution statement

**Manisha Joshi:** Writing – original draft, Methodology, Investigation, Conceptualization. **Gurvendra Pal Singh:** Writing – review & editing, Investigation. **Ipsheta Bose:** Writing – review & editing. **Tianxi Yang:** Writing – review & editing. **Azadeh Babaei:** Writing – review & editing. **Somesh Sharma:** Writing – review & editing, Validation, Supervision, Investigation. **Krishna Aayush:** Writing – review & editing, Supervision, Investigation.

## Declaration of competing interest

The authors declare that they have no known competing financial interests or personal relationships that could have appeared to influence the work reported in this paper.

## Data Availability

Data will be made available on request.

## Appendix A Supplementary data

Supplementary material Image 1
