# Supplementary material for: Development of antimicrobial nanoemulsion edible coating of xanthan gum incorporated with pomelo peel extract for cheese preservation
Source: Food Chem X. 2025 Jun 25;29:102692. doi: 10.1016/j.fochx.2025.102692 (PMC12270940; doi:10.1016/j.fochx.2025.102692)
Supplement: Supplementary file 1 — Supplementary material [file mmc1.docx]

**Development of fortified antimicrobial active nanoemulsionbased edible coating incorporating *Citrus grandis* peel extract for extending shelf life of cheese**

Manisha Joshi^1^, Gurvendra Pal Singh^2^, Ipsheta Bose^1,3^, Tianxi Yang^3^, Azadeh Babaei^4^, Somesh Sharma^5^* Krishna Aayush^6*^

^1^ *School of Bioengineering and Food Technology, Shoolini University, Bajhol, Distt Solan 173229, India*

*^2^**Department of Food Technology School of Engineering and Technology, Jaipur National University, Jaipur, Rajasthan, 302017, India*

*^3^ Food, Nutrition and Health, Faculty of Land and Food System, The University of British Columbia, Vancouver, BC V6T 1Z4, Canada*

*^4^* *Department of Chemistry, Karaj Branch, Islamic Azad University, Karaj, Iran*

*^5^ MS Swaminathan School of Agriculture, Shoolini University, Bajhol, Distt Solan 173229, India*

*^6^* *Department of food science and technology, Graphic Era (Deemed to be University),* *Dehradun, Uttarakhand, 248002 India*

* Corresponding author- Somesh Sharma: [someshsharma@shooliniuniversity.com](mailto:someshsharma@shooliniuniversity.com)

Krishna Aayush: [krishnaaayush2013@gmail.com](mailto:krishnaaayush2013@gmail.com)


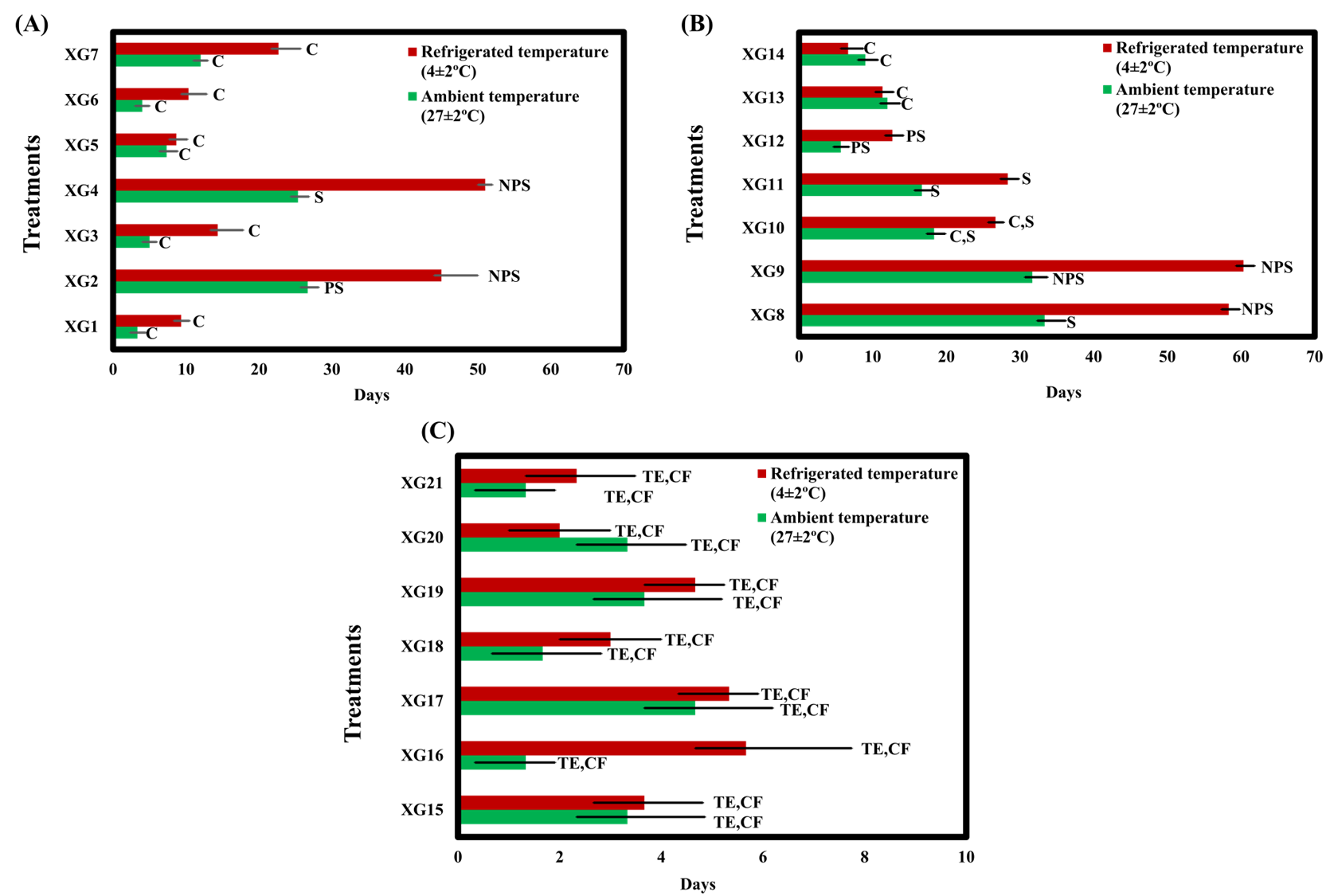


**Fig S1. Storage stability of nanoemulsion prepared using different concentrations of Xanthan Gum and Tween 80 during storage at different temperature storage conditions. (A) 0.1 % XG with 1-4% Tween 80; (B) 0.2% XG with 1-4% Tween 80; (C) 0.3% XG with 1-4% Tween 80. NPS: No phase separation; C: Creaming; S: Sedimentation; PS: Phase separation; TE: Thick emulsion; CF: Clump formation.**


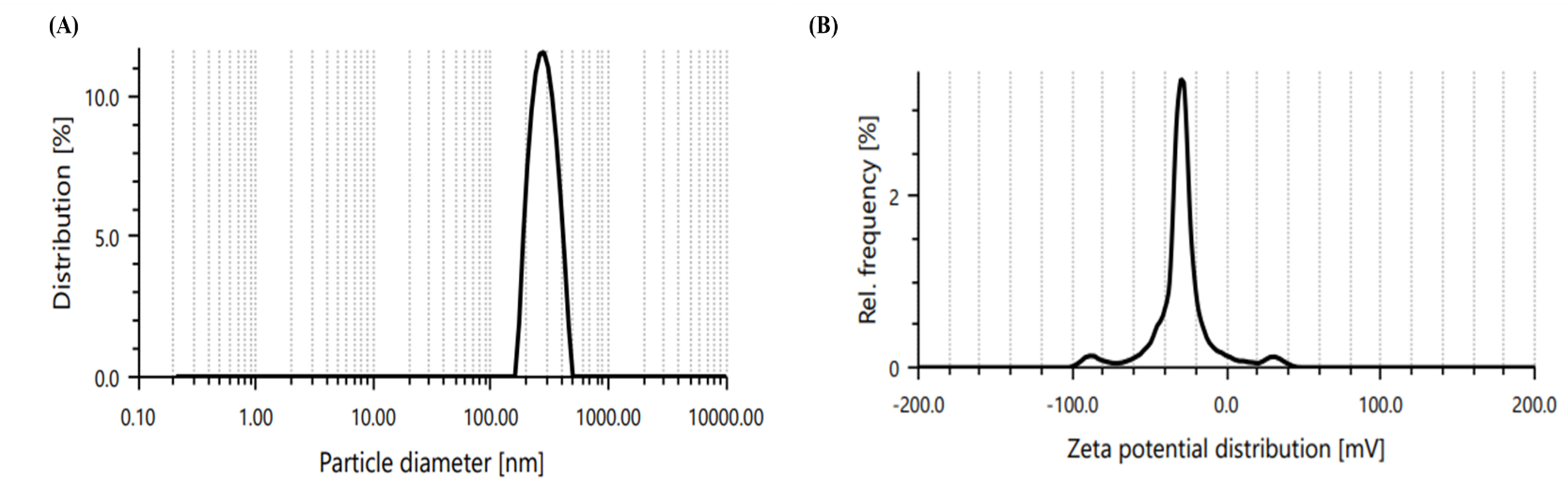


**Fig S2.** **PPE (pomelo peel extract) based nanoemulsion (A) Particle size through Dynamic Light Scattering (B) Zeta Potential**
